# Knowledge and Patterns Related to Nasal Decongestant Use Among the General Population in the Jazan Region of Saudi Arabia: A Cross-Sectional Study

**DOI:** 10.7759/cureus.90924

**Published:** 2025-08-25

**Authors:** Khalil I Kariri, Hadi M Mokarbesh, Abdulrahman A Otaif, Naif K Mahzara, Dhiyaa A Otayf, Rana A Sumayli, Futon A Akoor, Renad Madkhali, Waleed A Alabdali, Zakaria A Alhazmi

**Affiliations:** 1 Otolaryngology, King Fahd Central Hospital, Jazan, SAU; 2 Medicine, Jazan University, Jazan, SAU

**Keywords:** appropriate use, jazan region, nasal decongestants, saudi arabia, self-medication, usage patterns

## Abstract

Background: Although nasal decongestants are commonly used to treat respiratory symptoms, their inappropriate use may lead to complications such as rhinitis medicamentosa. There is a lack of research examining patterns of use and knowledge in Saudi Arabia, particularly in the Jazan Region.

Aim: This study aimed to evaluate the knowledge, patterns, and factors related to nasal decongestant use among adults in the Jazan Region of Saudi Arabia.

Methods: A cross-sectional study was conducted between January and June 2024. Data were collected through an online survey distributed via social media platforms. The validated Arabic online questionnaire collected data on the sociodemographic profile, patterns of use, and knowledge of nasal decongestants from 439 participants aged 18 years and above. Appropriate use was defined as obtaining the medication from healthcare facilities or using it for less than five days with a maximum of three doses per day. The data were analyzed descriptively and using bivariate and multivariate logistic regression models.

Results: The participants were predominantly young (average age, 27.8 years), female (65.4%), and university educated (67.9%). Of the 312 users, 57.1% demonstrated an appropriate duration of use (less than five days), and 42.9% exceeded the recommended duration of use. Regarding concerning patterns, 75% procured decongestants illegally from pharmacies and 25% self-medicated. There were significant knowledge gaps, with only 35.5% of participants aware of the rebound congestion risk and 43.1% incorrectly believing that decongestants were safe for all children. A strong dose-response relationship was observed between knowledge and appropriate use, with 32.1% and 71.2% for the low and high knowledge tertiles, respectively (p < 0.001). Employment in healthcare (adjusted odds ratio (AOR) = 2.45; p < 0.001) and postgraduate qualifications (AOR = 3.21; p = 0.003) were the strongest predictors of appropriate usage. The rate of complications significantly increased with a duration of use greater than seven days (risk ratio (RR) = 5.63; 95% CI: 2.17-14.59).

Conclusions: Significant knowledge gaps and patterns of inappropriate nasal decongestant use were observed among the residents of Jazan. There is a need for tailored educational strategies that address appropriate usage, duration, healthcare worker status, and raters with lower levels of educational attainment.

## Introduction

Nasal congestion is one of the most common symptoms observed in primary healthcare practices and is often associated with allergic and non-allergic rhinitis, acute rhinosinusitis, and nasal polyps [1]. This symptom is a consequence of nasal airway pathway obstruction, resulting in altered sensation and decreased quality of life. The diagnostic differentiation between anatomical nasal obstruction and dysfunctional nasal congestion can be effectively evaluated by the application of topical nasal decongestants [2].

As for nasal decongestants, the pharmacological category is frequently used in the field of otorhinolaryngology and medicine at large for reducing nasal congestion in upper respiratory tract infections, sinusitis, and allergic rhinitis. These sympathomimetic drugs can be administered systemically and topically and exert their activity by binding to adrenergic receptors. The most commonly prescribed formulations include oxymetazoline, xylometazoline, pseudoephedrine, and phenylephrine [3].

Imidazole compounds used in decongestants, such as xylometazoline and oxymetazoline, have become popular over-the-counter medications due to their marketing and rapid onset of action. These agents function post-synaptically by binding to alpha-2 adrenergic receptors, causing vasoconstriction and reducing blood flow to the turbinates, which positively increases the patency of the nasal passages. However, the therapeutic benefits may become counterproductive and harmful when used too liberally due to a paradoxical response via a negative feedback loop, which reduces norepinephrine release, leading to pseudoephedrine congestion and nasal swelling. Chronic use of nasal decongestants may result in rhinitis medicamentosa, characterized by congestion exacerbated by drug removal [4].

Rhinitis medicamentosa refers to adverse nasal congestion that develops due to the use of medications other than nasal sprays, including oral β-adrenoceptor antagonists, antipsychotics, oral contraceptives, and specific antihypertensive agents [5]. The ease of access to nasal decongestants and the unrestrained use of self-medication stem from the remarkable effectiveness of nasal decongestants in alleviating symptoms. It is especially concerning that nasal decongestants are included in over-the-counter oral fixed combinations with other medications, such as cetirizine, paracetamol, and ibuprofen, because this greatly increases the risk of misuse and abuse of such medicines. Although infrequently encountered, overdose situations may result in severe and potentially life-threatening central neurological effects, including stroke, seizures, and headaches, along with cardiovascular consequences such as palpitations, tachycardia, and hypertensive crises [6].

Although a wide range of nasal decongestant methods and products is available, understanding of their appropriate use, associated risks, and long-term implications remains inadequate, particularly among the general population. In Saudi Arabia, misuse of nasal decongestants appears to be a pressing concern, compounded by limited awareness of the available therapeutic options. Several studies have assessed knowledge levels and patterns of misuse across different contexts, and their findings consistently indicate low awareness and frequent inappropriate use [2,3,7,8].

The Jazan Region of Saudi Arabia presents unique healthcare and socioeconomic challenges that are likely to impact health literacy and medication adherence. Despite clear clinical implications regarding the proper use of nasal decongestants and the potential consequences of their misuse, no thorough investigations have examined their use, related sociodemographic factors, or attitudes among adults in this region. This gap poses a significant public health challenge, particularly given the easy availability of medications, common self-medication practices, and limited awareness of associated risks. Accordingly, this study aimed to assess knowledge and demographic determinants of nasal decongestant use among adults in the Jazan Region to inform evidence-based interventions for safer and more rational use.

## Materials and methods

Study design and setting

This community-based cross-sectional study was conducted in the Jazan Region, southwestern Saudi Arabia, from January to June 2024. The Jazan Region, with a population of approximately 1.6 million, comprises 16 governorates and features a unique and diverse socioeconomic and healthcare access landscape.

Study population and sampling 

The study population consisted of residents of the Jazan Region who were ≥18 years old and able to read Arabic. Using the Raosoft sample size calculator (Raosoft Inc., Seattle, WA, USA), we determined a minimum sample size of 385 participants, achieving a 95% confidence interval (CI) and a 5% margin of error. With a 10% non-response adjustment, the target sample size was set to 424. Given the limited resources and broad geographic diversity within the region, we employed online convenience sampling, which was a more cost-effective approach.

Data gathering tools

A validated Arabic questionnaire, along with a self-administered questionnaire, was used for this study [3]. A pilot study was conducted to assess the participants' clarity, comprehension, and completion within the given time constraints. Thirty participants were surveyed, all of whom were excluded from the final analysis. The final instrument comprised three sections. 

Section 1: Sociodemographic Characteristics (10 Items)

It included region of residence, age, gender, nationality, education level, marital status, monthly income, employment sector, and smoking status. 

Section 2: Patterns of Decongestant Use (12 Items)

It included history of use, duration, frequency, type of decongestant, reasons for use, concomitant medications, source of recommendation, place of acquisition, monthly consumption, symptom improvement, and diagnosed complications.

Section 3: Knowledge Assessment (11 Items)

It included awareness of efficacy, rebound congestion, safety in children, side effects, proper technique, administration, and contraindications. Responses could be classified as "correct," "incorrect," or "don't know."

Data collection procedures 

Data were gathered through an anonymous online survey created on Google Forms (Google LLC, Mountain View, CA, USA), which was shared on social media (WhatsApp, Telegram, Twitter/X) among the residents of Jazan. The survey was accessible for four months. The first page included the study details, objectives, and consent forms. Consent was required to access the questionnaire, and no personal identifiers were linked to the data to maintain the anonymity of the participants.

Study outcomes

The primary outcome was the knowledge score (0-9 points), derived from answering nine core knowledge items (with some items reverse-coded). 

Secondary outcomes included appropriate use, classified as acquisition from a healthcare facility or a duration of less than five days with a frequency of less than or equal to three times a day. Decongestant use patterns were classified based on duration, frequency, and source of use. 

Statistical analysis 

The data collected from Google Forms were first processed for cleaning and coding in Microsoft Excel (Microsoft Corp., Redmond, WA, USA). Data analysis was performed using SAS 9.4 (SAS Inc., Cary, NC, USA). For the descriptive analysis, frequencies and percentages were reported for categorical variables. Mean values accompanied by standard deviations are reported for normally distributed data, while skewed data are reported as medians with interquartile ranges. For the bivariate analysis, categorical variables were analyzed using the chi-squared test, and continuous variables were assessed using either independent t-tests or Mann-Whitney U tests. One-way analysis of variance (ANOVA) and Kruskal-Wallis tests were used to determine mean differences across several groups. 

Predictors of appropriate use were determined using multivariate logistic regression with bivariate analysis, where p < 0.20 served as the cutoff for entering variables into the model. Model fit was assessed using the Hosmer-Lemeshow test and Nagelkerke R². Multiple linear regression was used to determine the predictors of knowledge scores. Risk ratios (RRs) with 95% CIs were used to evaluate the relationship between complications and duration of use. All analyses were conducted at a significance level of p < 0.05.

Ethical considerations 

The study protocol was approved by the Local Committee for Research Ethics of Jazan University (approval number: REC-46/09/1421; date: March 5, 2025). All participants provided electronic informed consent. Participation was voluntary, and the participants were free to withdraw at any time. Data were securely stored and accessible only to the research team.

## Results

Sociodemographic characteristics

The study population (n = 439) was predominantly young (mean age, 27.8 years), female (65.4%), and well educated (67.9% with a university-level education). Most participants were single (56.0%), had a low-to-moderate income (<10,000 SAR: 75.4%), and were non-smokers (88.6%). The high proportion of students (37.6%) and healthcare workers (20.9%) suggests a relatively educated sample, which may influence the knowledge and usage patterns (Table 1).

**Table 1:** Sociodemographic characteristics of the study participants (n = 439) IQR: interquartile range

Characteristic	n (%)
Age (years)	
Mean ± SD	27.8 ± 10.2
Median (IQR)	25 (21-31)
Range	18-65
Gender	
Female	287 (65.4)
Male	152 (34.6)
Region	
Jazan	156 (35.5)
Abu Arish	89 (20.3)
Samtah	73 (16.6)
Sabya	52 (11.9)
Al-Darb	41 (9.3)
Other	28 (6.4)
Education level	
University (bachelor/diploma)	298 (67.9)
High school	86 (19.6)
Postgraduate	35 (7.9)
Primary/intermediate	20 (4.6)
Marital status	
Single	246 (56.0)
Married	178 (40.6)
Divorced/widowed	15 (3.4)
Monthly income (SAR)	
<5,000	189 (43.1)
5,000-9,999	142 (32.3)
10,000-14,999	73 (16.6)
≥15,000	35 (8.0)
Employment sector	
Student	165 (37.6)
Healthcare	92 (20.9)
Education	78 (17.8)
Private sector	56 (12.8)
Unemployed	48 (10.9)
Smoking status	
Non-smoker	389 (88.6)
Current smoker	50 (11.4)

Among the users (n = 312), most demonstrated an appropriate duration of use (<5 days: 57.1%), although 42.9% exceeded the recommendations. Otrivin was the predominant brand (50.0%). The findings revealed that 25% of the participants self-medicated without consultation, 75% obtained decongestants from pharmacies without a prescription, and 14.4% used ≥3 bottles per month, suggesting potential overuse. The 8% complication rate warrants attention (Table 2).

**Table 2:** Pattern of nasal decongestant use among participants who have used decongestants (n = 312) *Participants were allowed to choose more than one choice. The percentage is calculated based on the total number of participants who use nasal decongestants (n = 312)

Variable	n (%)
Duration of use	
Less than 5 days	178 (57.1)
5-7 days	76 (24.4)
More than 7 days	58 (18.6)
Daily use frequency	
On symptoms only	142 (45.5)
1-2 times per day	89 (28.5)
3 times per day	53 (17.0)
>3 times per day	28 (9.0)
Type of decongestant	
Otrivin	156 (50.0)
Vicks Sinex	73 (23.4)
Afrin	45 (14.4)
Other	38 (12.2)
Reason for use*	
Nasal congestion	198 (63.5)
Common cold	167 (53.5)
Allergic rhinitis	89 (28.5)
Sinusitis	42 (13.5)
Other	23 (7.4)
Source of recommendation	
Pharmacist	134 (42.9)
Self-medication	78 (25.0)
Physician	62 (19.9)
Family/friends	38 (12.2)
Source of obtaining a decongestant	
Pharmacy	234 (75.0)
Hospital/clinic	56 (17.9)
Supermarket	22 (7.1)
Bottles used per month	
One	189 (60.6)
Two	78 (25.0)
Three or more	45 (14.4)
Symptom improvement	
Yes	256 (82.1)
No	56 (17.9)
Diagnosed complications	
No	287 (92.0)
Yes	25 (8.0)

Knowledge assessment

The knowledge gaps were substantial, as shown in Table 3. Only 35.5% were aware of the rebound congestion risk, and 44.2% were unaware. Misconceptions were common: 43.1% incorrectly believed that decongestants were safe for all children, and 24.4% were unaware of the risks of sharing infections. Better knowledge was shown for symptom relief (77.9% correct) and administration technique (71.1% correct), indicating selective awareness focused on benefits rather than risks.

**Table 3:** Knowledge assessment regarding nasal decongestant use (n = 439) *Indicates reverse-scored items where "No" is the correct answer

Knowledge item	Correct n (%)	Incorrect n (%)	Don't know n (%)
Nasal decongestants can relieve cold symptoms	342 (77.9)	31 (7.1)	66 (15.0)
Use for >5 days can cause rebound congestion	156 (35.5)	89 (20.3)	194 (44.2)
Should be used for symptom relief only	267 (60.8)	78 (17.8)	94 (21.4)
Safe for all children	142 (32.3)*	189 (43.1)	108 (24.6)
Topical decongestants are ineffective due to poor absorption	98 (22.3)*	234 (53.3)	107 (24.4)
Sharing bottles poses no infection risk	87 (19.8)*	298 (67.9)	54 (12.3)
Major cause of poisoning in children <5 years	178 (40.5)	67 (15.3)	194 (44.2)
Best position: tilt the head forward when administering	312 (71.1)	45 (10.3)	82 (18.7)
Saline is an effective adjunct treatment	289 (65.8)	56 (12.8)	94 (21.4)

Knowledge of side effects and contraindications

**Figure 1:**
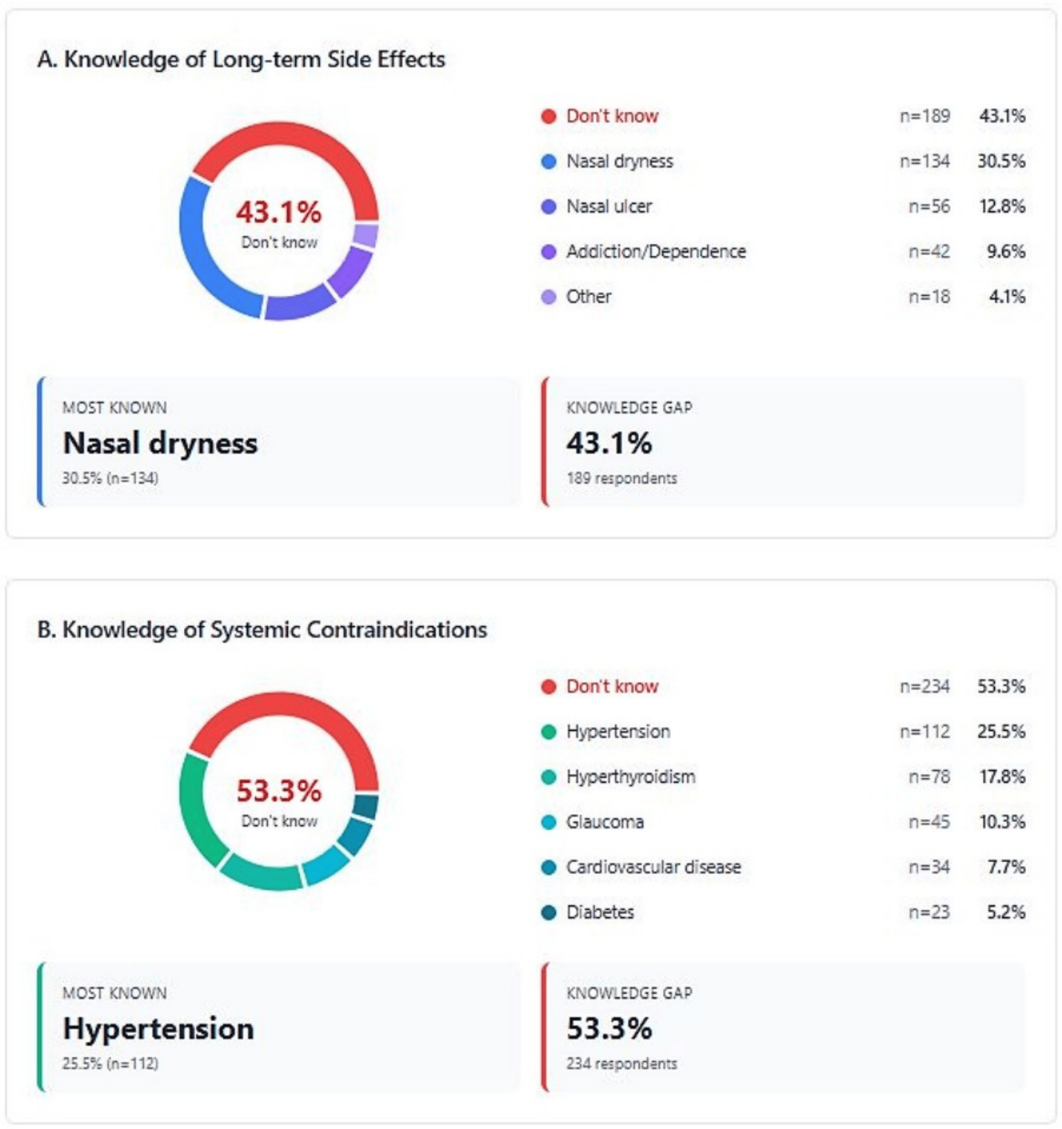
Knowledge of side effects and contraindications (n = 439)

Critical knowledge deficits are shown in Figure 1. Of the respondents, 43.1% were unaware of any long-term side effects, and 53.3% were unaware of contraindications. Among those with knowledge, nasal dryness (30.5%) was the most recognized side effect, but serious effects such as addiction (9.6%) were poorly known. Only 25.5% of the respondents knew that hypertension is a contraindication, despite its importance for patient safety.

Education emerged as the strongest predictor (Table 4). Postgraduate education tripled the odds of appropriate use (adjusted odds ratio (AOR) = 3.21; p = 0.003). Healthcare employment (AOR = 2.45; p < 0.001) and professional consultation (AOR = 2.12; p = 0.004) increased the likelihood of appropriate usage. Age >35 years (AOR = 1.89; p = 0.012) and female gender (AOR = 1.54; p = 0.044) showed modest associations. The model's good fit (R² = 0.287) confirmed that these factors explained a substantial variance in appropriate use patterns.

**Table 4:** Factors associated with appropriate nasal decongestant use: multivariate logistic regression analysis Model fit: Hosmer-Lemeshow test p = 0.412; Nagelkerke R² = 0.287

Variable	Crude OR (95% CI)	P-value	Adjusted OR (95% CI)	P-value
Age				
18-25 years	1.00 (reference)	-	1.00 (reference)	-
26-35 years	1.45 (0.98-2.14)	0.062	1.38 (0.91-2.09)	0.126
>35 years	2.12 (1.34-3.35)	0.001	1.89 (1.15-3.11)	0.012
Gender				
Male	1.00 (reference)	-	1.00 (reference)	-
Female	1.67 (1.12-2.49)	0.011	1.54 (1.01-2.35)	0.044
Education level				
High school or less	1.00 (reference)	-	1.00 (reference)	-
University	2.34 (1.52-3.61)	<0.001	2.18 (1.38-3.44)	<0.001
Postgraduate	3.67 (1.76-7.65)	<0.001	3.21 (1.49-6.92)	0.003
Employment in healthcare				
No	1.00 (reference)	-	1.00 (reference)	-
Yes	2.89 (1.82-4.59)	<0.001	2.45 (1.51-3.98)	<0.001
Previous advice on use				
No	1.00 (reference)	-	1.00 (reference)	-
Yes	2.23 (1.49-3.34)	<0.001	1.98 (1.29-3.04)	0.002
Source of recommendation				
Self-medication	1.00 (reference)	-	1.00 (reference)	-
Healthcare professional	2.56 (1.58-4.15)	<0.001	2.12 (1.27-3.54)	0.004
Family/friends	0.78 (0.41-1.49)	0.452	0.89 (0.45-1.76)	0.734

Knowledge-behavior association

A strong dose-response relationship emerged between knowledge and appropriate use, as shown in Table 5. Only 32.1% of those with low knowledge (0-3 correct) used decongestants appropriately, compared to 71.2% of those with high knowledge (7-9 correct). A significant trend (χ² = 38.45; p < 0.001) was observed, demonstrating that knowledge directly translated to better practice, with a 2.2-fold increase in appropriate use between the lowest and highest knowledge tertiles.

**Table 5:** Association between knowledge score and decongestant use patterns Chi-squared test for trend: χ² = 38.45; p < 0.001

Knowledge score tertile	Appropriate use n (%)	Inappropriate use n (%)	P-value
Low (0-3 correct)	45 (32.1)	95 (67.9)	<0.001
Medium (4-6 correct)	78 (54.2)	66 (45.8)	
High (7-9 correct)	89 (71.2)	36 (28.8)	

Knowledge score differences by demographics

Healthcare workers showed the largest knowledge advantage (5.8 vs. 4.3; p < 0.001), followed by differences in education level. The progressive increase from high school (3.8) to university (4.7) to postgraduate education (5.9) (p < 0.001) suggests that formal education has a strong influence on knowledge of medication. Females scored modestly higher than males (4.8 vs. 4.2; p = 0.018), and knowledge increased with age, indicating experiential learning (Table 6).

**Table 6:** Comparison of knowledge scores by demographic characteristics Analysis: independent t-test for two groups; one-way ANOVA for multiple groups ANOVA: analysis of variance

Characteristic	Mean knowledge score ± SD	P-value
Gender		
Male	4.2 ± 2.1	0.018
Female	4.8 ± 2.3	
Age group		
18-25 years	4.3 ± 2.2	0.004
26-35 years	4.9 ± 2.1	
>35 years	5.2 ± 2.3	
Education level		
High school or less	3.8 ± 2.0	<0.001
University	4.7 ± 2.2	
Postgraduate	5.9 ± 2.1	
Healthcare worker		
No	4.3 ± 2.1	<0.001
Yes	5.8 ± 2.2	

Predictors of knowledge (multiple regression)

The model explained 34.2% of the variance in the knowledge (R² = 0.342). Healthcare employment (B = 1.23) and postgraduate education (B = 1.67) were the strongest predictors, each adding more than one point to the knowledge scores. Every year of age added 0.04 points (p < 0.001), and receiving professional advice added 0.92 points. The cumulative effect suggests that targeted education could substantially improve knowledge; a healthcare worker with postgraduate education scored approximately 3 points higher than the baseline (Table 7).

**Table 7:** Predictors of knowledge about nasal decongestants: multiple linear regression analysis Model summary: R² = 0.342; adjusted R² = 0.331; F(7,431) = 32.04; p < 0.001

Variable	B	SE	β	t	P-value	95% CI for B
(Constant)	2.34	0.42	-	5.57	<0.001	1.52, 3.16
Age (years)	0.04	0.01	0.18	3.89	<0.001	0.02, 0.06
Female gender	0.56	0.21	0.12	2.67	0.008	0.15, 0.97
University education	0.89	0.24	0.19	3.71	<0.001	0.42, 1.36
Postgraduate education	1.67	0.38	0.21	4.39	<0.001	0.92, 2.42
Healthcare worker	1.23	0.26	0.23	4.73	<0.001	0.72, 1.74
Previous decongestant use	0.78	0.22	0.16	3.55	<0.001	0.35, 1.21
Received advice on use	0.92	0.23	0.19	4.00	<0.001	0.47, 1.37

Duration-complication relationship

A clear dose-response pattern emerged, as shown in Table 8. Complications increased from 3.4% (<5 days) to 10.5% (5-7 days) to 19.0% (>7 days). Users exceeding seven days had 5.63 times higher risk of complications (95% CI: 2.17-14.59; p < 0.001). The tripling of risk with 5-7 days of use (RR = 3.11) suggests that complications begin early, supporting strict adherence to the five-day limit.

**Table 8:** Association between duration of use and development of complications Chi-squared for trend: χ² = 15.68; p < 0.001

Duration of use	Complications n (%)	No complications n (%)	RR (95% CI)	P-value
<5 days	6 (3.4)	172 (96.6)	1.00 (reference)	-
5-7 days	8 (10.5)	68 (89.5)	3.11 (1.11-8.73)	0.024
>7 days	11 (19.0)	47 (81.0)	5.63 (2.17-14.59)	<0.001

Knowledge varies by use patterns

Appropriate users consistently showed higher knowledge across all dimensions, as shown in Table 9. The 2.5-point knowledge deficit among those obtaining decongestants from non-healthcare sources (3.2 vs. 5.7; p < 0.001) highlights the importance of professional guidance. Regular daily users scored 1.1 points lower than as-needed users, suggesting that poor knowledge drives habitual use. These 1-2-point differences represent 20-25% knowledge gaps with direct clinical implications.

**Table 9:** Subgroup analysis of knowledge by decongestant use patterns

Subgroup	n	Mean knowledge score ± SD	Mean difference (95% CI)	P-value
Duration of use				
Appropriate (<5 days)	178	5.2 ± 2.1	Reference	-
Inappropriate (≥5 days)	134	4.1 ± 2.2	-1.1 (-1.6, -0.6)	<0.001
Frequency of use				
As needed only	142	5.3 ± 2.0	Reference	-
Regular daily use	170	4.2 ± 2.3	-1.1 (-1.6, -0.6)	<0.001
Source of obtaining				
Healthcare facility	56	5.7 ± 1.9	Reference	-
Pharmacy	234	4.6 ± 2.2	-1.1 (-1.7, -0.5)	<0.001
Other sources	22	3.2 ± 2.1	-2.5 (-3.4, -1.6)	<0.001

## Discussion

This study identified concerning habits regarding the use of nasal decongestants in the Jazan Region of Saudi Arabia. It was found that 42.9% of the participants reported using nasal decongestants for longer than the recommended duration and 35.5% were aware of the potential for rebound congestion. Most importantly, the study demonstrated a robust correlation between knowledge and appropriate use, with high-knowledge participants exhibiting a 71.2% appropriate use rate compared with 32.1% among those with low knowledge. Employment in healthcare and holding a postgraduate degree were the strongest predictors of appropriate use, indicating that exposure to the profession significantly impacts behavior concerning medication safety.

Moreover, our results are consistent with the global pattern of decongestant abuse, where 31.9-49% of users exceed the recommended duration of use in all developed countries [9,10]. A 2022 national study found the prevalence of nasal decongestant use to be 45.1%, while 70.8% of these participants were using them for less than five days [2]. In a study conducted by Mokhatrish et al., only 15% of pharmacists believed that a physician's prescription was required to obtain nasal decongestants, while 87.3% believed that less than five days is the maximum safe duration for using these medications [7].

In the Al-Qunfudah Region, a 2023 study reported an even higher utilization rate of 76.6%. However, only 12.9% and 33.2% of participants correctly identified nasal dryness and mucosal ulceration as side effects of prolonged use. More than half (55.1%) were unaware of the term "rebound congestion," and the majority (84.6%) ignored contraindications [3]. In another region, Almutairi et al. reported widespread knowledge gaps regarding addiction potential (78.7%), appropriate duration of use (74.8%), side effects (83.4%), and risk of rebound congestion (78.4%) [8].

At the international level, European data show distinct age-related trends. Italian pharmacy surveys identified two peak user groups: younger adults aged 18-30 years and older adults aged 60-75 years. Estimates from Germany suggest that at least 100,000 people are affected by nasal decongestant addiction, though experts believe the true figure is closer to one million [9].

Nevertheless, the Jazan area demonstrates a significantly higher rate of pharmacy-sourced medications (75% without prescriptions) than the national Saudi average of 21% for pharmacist-recommended purchases [2,10]. This difference may indicate a regional pattern of healthcare access as an underlying factor, highlighting the important role of community pharmacies in ensuring the safety of medications. The 8% complication rate and the 5.63-fold greater risk associated with use after seven days are consistent with the international literature, which shows that rhinitis medicamentosa can occur as early as three days of continuous use, with a population incidence of 1-9% [11].

Demographic analysis revealed an equal sex distribution for the development of rhinitis medicamentosa, contradicting earlier assumptions about sex-based risk factors. Young and middle-aged adults face the highest risk, with adolescents being particularly vulnerable to developing dependency patterns. The speed of addiction development is the most concerning factor. Pathophysiological changes, including alpha-adrenergic receptor upregulation, can occur as early as three days of continuous use, although they typically manifest within 3-8 weeks [12,13].

The knowledge-behavior correlation (32.1% versus 71.2% for appropriate use) mirrors findings from previous health literacy and adherence correlations within the range of r = 0.14-0.342 [14-16]. Most troubling is the 43.1% prevalence of the misconception that nasal decongestants are safe for all children, which poses a risk of harmful pediatric health outcomes. This finding is similar to global research that revealed 25-37% of study participants had considerable knowledge gaps regarding the safety of over-the-counter medications for certain vulnerable populations [17-19]. Employment in healthcare and graduate education, as predictors of interest in these areas, align with international findings that exposure to the medical field is associated with a more accurate understanding and, paradoxically, higher rates of self-medication [20-22].

The strengths of this study include the significant sample size from a sparsely studied area and the use of validated tools to measure knowledge-behavior relationships. However, several limitations merit consideration. The cross-sectional design prevents causal inference, and reliance on self-reported questionnaires may introduce recall and social desirability bias. The exclusive use of online sampling could have limited participation from less digitally literate groups, while reliance on written questionnaires without face-to-face validation and possible language barriers may have added bias. The geographical focus on the Jazan Region restricts generalizability, and government strategies addressing health literacy, as well as broader community inclusion, should be considered in future studies. Finally, the study did not address the acute presentation of chronic conditions such as rhinitis medicamentosa and its long-term complications.

The identified knowledge gaps and resource misuse warrant immediate attention in public health. Comprehensive educational programs achieve a 25% reduction in misuse, whereas pharmacy-based interventions result in a 62% reduction [14,23,24]. Because of the strong relationship between education and behavior, targeted interventions should focus on counseling at the point of sale in pharmacies, where 75% of patients fill their prescriptions [2]. Labeling of rebound congestion risks and limits should be made more conspicuous. Healthcare providers should also be taught effective counseling skills and digital health tools, which have the potential to reduce errors by 54-87% [21,25]. Further studies should assess the impact of culturally tailored educational programs in the Jazan Region, evaluate pharmacy-based screening program protocols, and analyze the Saudi healthcare system regulatory frameworks to balance medication access and safety [23,26].

## Conclusions

This investigation revealed concerning knowledge deficits and misuse of nasal decongestants among adults in the Jazan Region of Saudi Arabia. While many users adhered to recommended duration limits, major gaps remained regarding rebound congestion and medication safety in children, emphasizing the need for targeted education. Postgraduate education and healthcare employment were the strongest predictors of appropriate use, whereas obtaining decongestants without prescriptions was associated with increased complications and highlighted issues of access, counseling, and regulatory oversight. These findings call for pharmacist training, culturally tailored patient education, and stricter regulation. Prescription education should stress duration limits, rebound risk, and contraindications, while further research is needed to assess specific strategies. The strong correlation between knowledge and behavior supports that well-designed education can improve safety, prevent rhinitis medicamentosa, and rationalize decongestant use.
